# Analysis of structural effects of sickle cell disease on brain vasculature of mice using three-dimensional quantitative phase imaging

**DOI:** 10.1117/1.JBO.28.9.096501

**Published:** 2023-09-09

**Authors:** Caroline Elizabeth Serafini, Hannah Song, Manu O. Platt, Francisco E. Robles

**Affiliations:** a Georgia Institute of Technology, George W. Woodruff School of Mechanical Engineering, Atlanta, Georgia, United States; b Georgia Institute of Technology, Wallace H. Coulter Department of Biomedical Engineering, Atlanta, Georgia, United States; c National Institute of Biomedical Imaging and Bioengineering, National Institutes of Health, Bethesda, Maryland, United States

**Keywords:** phase microscopy, label-free imaging, sickle cell disease, cerebral vasculature

## Abstract

**Significance:**

Although the molecular origins of sickle cell disease (SCD) have been extensively studied, the effects of SCD on the vasculature—which can influence blood clotting mechanisms, pain crises, and strokes—are not well understood. Improving this understanding can yield insight into the mechanisms and wide-ranging effects of this devastating disease.

**Aim:**

We aim to demonstrate the ability of a label-free 3D quantitative phase imaging technology, called quantitative oblique back-illumination microscopy (qOBM), to provide insight into the effects of SCD on brain vasculature.

**Approach:**

Using qOBM, we quantitatively analyze the vasculature of freshly excised, but otherwise unaltered, whole mouse brains. We use Townes sickle transgenic mice, which closely recapitulate the pathophysiology of human SCD, and sickle cell trait mice as controls. Two developmental time points are studied: 6-week-old mice and 20-week-old mice. Quantitative structural and biophysical parameters of the vessels (including the refractive index (RI), which is linearly proportional to dry mass) are extracted from the high-resolution images and analyzed.

**Results:**

qOBM reveals structural differences in the brain blood vessel thickness (thinner for SCD in particular brain regions) and the RI of the vessel wall (higher and containing a larger variation throughout the brain for SCD). These changes were only significant in 20-week-old mice. Further, vessel breakages are observed in SCD mice at both time points. The vessel wall RI distribution near these breaks, up to 350  μm away from the breaking point, shows an erratic behavior characterized by wide RI variations. Vessel diameter, tortuosity, texture within the vessel, and structural fractal patterns are found to not be statistically different. As with vessel breaks, we also observe blood vessel blockages only in mice brains with SCD.

**Conclusions:**

qOBM provides insight into the biophysical and structural composition of brain blood vessels in mice with SCD. Data suggest that the RI may be an indirect indicator of vessel rigidity, vessel strength, and/or tensions, which change with SCD. Future *ex vivo* and *in vivo* studies with qOBM could improve our understanding of SCD.

## 1 Introduction

Sickle cell disease (SCD) is a genetic disorder characterized by hemoglobin polymerization under deoxygenated conditions that alters red blood cell (RBC) morphology, their oxygen-carrying capacity, and lysing, prematurely leading to anemia. Sickling of RBCs causes membrane damage that can lead to RBC aggregation and adhesion to the vascular wall, which can cause vaso-occlusive crises, strokes, and acute chest syndrome.^1^^,^^2^ There is also a growing body of evidence showing that SCD causes chronic inflammation, increased oxidative stress, and other abnormalities that lead to a wide variety of vascular pathologies and consequential organ damage and complications.^3^ However, these effects of SCD on the vasculature are not well understood. A significant challenge in gaining a deeper understanding of the interaction between RBC alterations and phenotypical changes in the vasculature is a lack of imaging tools available to capture physiologically relevant changes at the cellular and tissue scales *in situ* or *in vivo*.

Clots and blockages due to SCD have primarily been captured through magnetic resonance imaging,^4^ computed tomography,^5^ and ultrasound imaging.^1^ These imaging modalities can provide a broad view of patients’ symptoms and can help localize strokes and ischemia; however, they lack critical histology-grade information that can lead to a better understanding of the underlying mechanisms of blood cell adhesion, stroke, and more.^5^^–^^10^ Recently, optical imaging methods have been employed; these methods have the advantage of providing higher spatial resolution to view these tissue structures in greater detail. Studies have used fluorescence-based methods to study, for instance, the interactions between brain endothelial cells and RBCs exposed to oxidative stress *in vitro*^11^ or to monitor SCD treatments *in vivo* using a dorsal skin-fold window chamber model.^12^ However, these studies use fluorescent labels, which have been shown to impact the blood chemistry and physiology of the blood through vasodilatation and osmolarity of the vessels.^13^ Further, fluorescence microscopy suffers from phototoxicity and photobleaching and can only provide contrast for a few labeled targets of interest. Thus, there is a significant need for a method that can provide access to both the morphology of blood cells and the structure and composition blood vessels at the cellular and tissue levels to study their interactions.

In this work, we apply quantitative oblique back-illumination microscopy (qOBM) to study the effects of SCD on the vasculature of fresh, whole, mouse brains. qOBM is a label-free imaging technique that enables tomographic (3D) quantitative phase imaging (QPI) of thick scattering samples with epi-illumination.^14^^,^^15^ As a label-free imaging technique, qOBM provides unique access to the morphology of blood vessels and blood cells within, in addition to their refractive index (RI) composition, which is linearly proportional to dry mass. Here, we assess the structural and biophysical differences between fresh whole mouse brains of control mice heterozygous for the sickle cell allele (referred to as AS mice with sickle cell trait) and mice homozygous with two sickle cell alleles (referred to as SS mice with SCD) at two ages: 6-week-old mice at the adolescent stage and 20-week-old mice at the more mature adult stage. Results show significant differences between the AS and SS mice, with SS mice possessing blood vessel walls that are thinner around the circle of Willis (CoW) and with overall (i.e., independent of the anatomical location in the brain) higher RI values and variations. The observed RI changes are, however, age-dependent, with results showing statistically significant differences between the AS and SS groups only in the 20-week-old mice. Further, vessel breakages are observed around the CoW in SS 6- and 20-week-old mice, and the vessel wall RI distribution near breaks, up to 350  μm away from the breaking point, show an erratic behavior characterized by wide RI fluctuations.

This work uses *ex vivo* freshly excised, whole brains as a proof of concept, but the approach is also well suited for *in vivo* studies, including animal models and potentially in humans using accessible anatomical locations. This approach has the potential to improve our understanding of SCD and its effect on the vasculature.

## 2 Materials and Methods

### 2.1 Animal Preparation

Townes sickle transgenic mice breeding pairs (B6; 129-Hbatm1(HBA) Tow Hbbtm2 (HBG1, HBB*) Tow/Hbbtm3 (HBG1, HBB) Tow/J) were obtained from The Jackson Laboratory. The animals were kept in climate-controlled rooms with a 12 h light-dark cycle and were allowed access to food and water *ad libitum*. Mice received Lab Diet 5001 after weaning and Lab Diet 5015 for breeding. Bed-o’Cobs ¼” cage bedding from The Andersons lab bedding was used. Newborn mice were evaluated for sickle status at weaning (21 days old) using blood hemoglobin gel analysis. Mice were euthanized using ${\mathrm{CO}}_{2}$ inhalation at 6-weeks-old or 20 weeks-old, followed by trans-cardiac perfusion with 60 ml of heparinized saline per mouse using a syringe pump at a constant flow rate (70  μl/min). The skin and skull were removed, and the brains were carefully excised and placed in a 50 ml tube of cold saline. All experiments were approved by Georgia Institute of Technology’s Institutional Animal Care and Use Committee. A total of 15 mouse brains were used in these sets of experiments: four 6-week-old SS mice, four 6-week-old AS mice, three 20-week-old SS mice, and three 20-week-old AS mice. The AS mice were used as controls.

### 2.2 qOBM Imaging

**Fig. 1:**
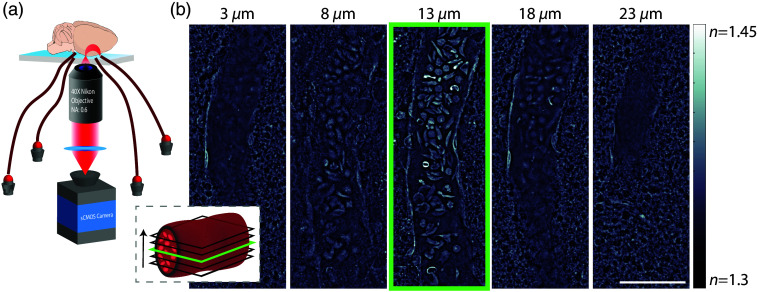
(a) Schematic of the qOBM imaging system consisting of an inverted brightfield microscope with epi-illumination. The sample (mouse brain) is sequentially illuminated by four optical fibers connected to 720 nm LEDs, followed by quantitative phase recovery. (b) A schematic demonstrating qOBM’s cross sectional capabilities of a blood vessel filled with cells highlighting the tomographic sectioning capabilities of the system, which can be applied to select the widest part of the vessel as indicated in green. Scale bar is 50  μm, and the gray-scale color map represents the RI properties (denoted with the variable n).

The qOBM imaging system consists of a conventional inverted brightfield microscope geometry with a few additions to enable tomographic phase imaging with epi-illumination.^14^^–^^18^ Unlike transmission based QPI, qOBM illuminates the samples in epi-mode with four 720 nm light-emitting diode (LED) light sources coupled to multimode fibers, each arranged 90-deg from one another around the microscopy objective [see Fig. 1(a)]. Light from a single LED is deployed through the multimode fiber into the thick sample (here, the brain), where it undergoes multiple scattering events. This results in some photons changing their trajectory to effectively create a virtual light source within the scattering object, emulating a transmission microscope. Further, on average, the light at the focal plane has an oblique, non-symmetric distribution; this process is known as oblique back-illumination.^19^ Based on the RI distribution of the tissue at the focal plane, oblique light is refracted toward or away from the acceptance angles of the microscope objective, which translates changes in the RI of the sample into intensity changes that can be detected by the camera without a dedicated interferometer. Two intensity images from opposing illumination angles are acquired and then subtracted to produce a qualitative differential phase contrast (DPC) image, with an observed shear angle along the direction of the sources.^19^ To recover quantitative phase information with qOBM, a pair of orthogonal DPC images are acquired and then deconvolved with the system’s optical transfer function via a Tikhonov regularized deconvolution, as previously described by Refs. 14, 15, and 18. After the deconvolution, qOBM yields clear cellular and sub-cellular contrast with quantitative image values reflective of the RI properties of the sample, denoted with the variable n. Figure 1(b) shows representative qOBM images of the brain vasculature of mice. We highlight that qOBM yields high-contrast and quantitative cross sectional images of the vessels, lumen, blood cells within, and other microscopic structures in the surrounding tissue. In this work, we use a 40×, 0.6 NA objective (Nikon S Plan Fluor LWD) and a CMOS camera (pco.edge 4.2 LT). The lateral resolution is 0.75  μm, and the axial resolution is 3.2  μm.

### 2.3 Refractive Index Calculations

qOBM provides quantitative phase information of the optical fields, which encodes the RI properties of cells and tissues. The relationship between the measured optical phase and the sample’s RI is given as

$$\Delta n=\frac{\lambda \Delta \phi }{2\pi \Delta z},$$ (1)

where $\Delta n=n_{o}-n_{m}$ represents the RI difference between the object ($n_{o}$) and the surrounding medium ($n_{m}$), λ represents the wavelength, Δϕ represents the phase difference, and Δz represents the thickness of the object, or in this case, the effective thickness of the slice provided by this 3D imaging method (i.e., the axial resolution of 3.2  μm).^14^ Here we take the medium’s RI ($n_{m}$) in the mouse brain to be 1.3440.^20^

### 2.4 Imaging Procedure with qOBM

To study the impact of SCD on vasculature, we first focused on the arteries of particular biological interest in SCD, including the CoW and middle cerebral artery (MCA) in the inferior side of the brain.^21^ These structures are the major sites of blockages that cause strokes in children with SCD.^21^^,^^22^ For each perfused and excised brain, qOBM images were taken from the surface up to ∼160  μm into the tissue along the CoW. This included imaging ∼1500  μm along the anterior cerebral artery (ACA), the internal carotid artery (ICA), and the MCA.

Next, we switch focus to the cortex of the brain on the superior side, which is an area accessible *in vivo* through a window-chamber model. Additionally, these vessels play an important role in cerebrovascular diseases and crisis events.^2^^,^^23^ As such, we imaged the superior side of the brain where we surveyed five separate regions that offered clear views of blood vessels in the cerebral cortex. Again, z-stacks were acquired from 0 to 160  μm at each region with a step size of Δz=1  μm.

### 2.5 Image Segmentation

**Fig. 2:**
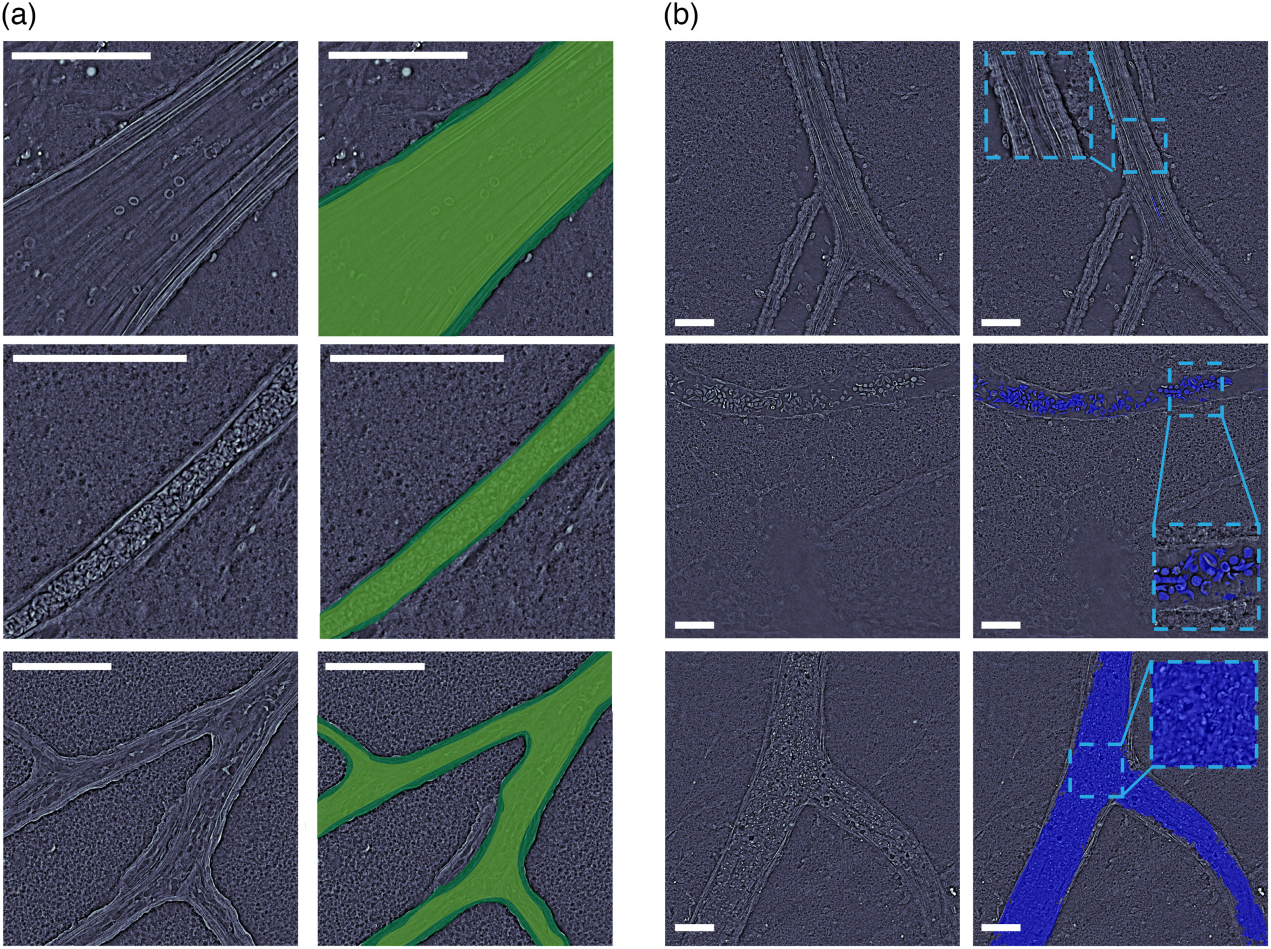
Representative qOBM images illustrating segmentation and calculations. (a) Segmentation of the interior vessel (light green) and the vessel wall (dark green). Examples include regions with a blood vessel largely void of blood cells (top), a vessel completely filled with blood cells (middle), and a vessel containing the junction of multiple vessels (bottom). (b) Blood vessel segmentation performed to calculate the percent fill of a vessel, showing a largely empty vessel (top), a vessel in which individual blood cells can be seen (middle), and a blood vessel packed tightly with blood cells (bottom). Scale bars are 100  μm.

Vessels were manually segmented and analyzed at the widest point of the vessel lumen (i.e., the center axial slice of the lumen). As illustrated in Fig. 1(b), as we image through the vessel axially, we can localize the center of the lumen based on the plane with the widest vessel diameter [indicated in green in Fig. 1(b)]. In the cortex, we were able to visualize arterioles, capillaries, and veins. In this work, we only included blood vessels with a diameter >20  μm due to physiological differences, including those in volume capacity and elasticity as exhibited by Xiong et al. and Ghanavati et al.^24^^,^^25^ This was done to ensure that the vessels analyzed were similar in composition and to exclude small capillaries with different physiological and mechanical properties. Manual segmentation of the blood vessel interior and the blood vessel wall was completed as seen in Fig. 2(a), without explicit knowledge of the sample type (sickle genotype or age). Following the interior vessel segmentation, edge detection was performed with Canny edge thresholding,^26^ and segmentation was used to separate the blood cells within the vessel [Fig. 2(b)]. Using the overall area of the vessel and the area of the blood cells segmented, a percentage fill of the blood vessel was calculated as $\%\text{Fill}=(A_{\text{Cells}}/A_{\text{Vessel}})*100$. Here $A_{\text{Cells}}$ represents the area of the blood cells in the vessel, and $A_{\text{Vessel}}$ represents the area of the entire blood vessel. Examples of this segmentation with empty, partially filled, and completely filled vessels can be seen in Fig. 2(b).

With the segmented image, the Euclidean distance from the center of the blood vessel to the outer boundary was calculated to determine the diameter of the vessel, not including the vessel wall thickness. Examples of this segmentation can be seen in Fig. 2(a). Additional measured parameters included blood vessel tortuosity, fractal morphological shape patterns,^27^ blood vessel texture analysis,^28^ and the angle that the MCA intersected the CoW; however, these parameters were found not to be statistically different between SS mice and the AS group. Results for these non-significant parameters are presented in the Sec. 1 in the Supplementary Material (Fig. S1 in the Supplementary Material) and will not be elaborated on further in the main text.

### 2.6 Statistical Analysis

Experimental conditions were repeated with a minimum of three biological replicates (see animal preparation section). All data presented in the graphs are shown as mean value and with error bars of standard deviation. Unpaired t-tests were used to determine the statistical significance (P<0.05) between experimental groups. For the statistical tests, the average values of each individual animal were taken to be the independent observation (i.e., N=4, 6-week-old AS mice; 4, 6-week-old SS mice; 3, 20-week-old AS mice; and 3, 20-week-old SS mice) unless otherwise noted.

## 3 Results and Discussion

### 3.1 CoW Vessels of 20-Week-old SS Mice Exhibit Thinner Vessel Walls, a Higher RI, and Increased Breakages

**Fig. 3:**
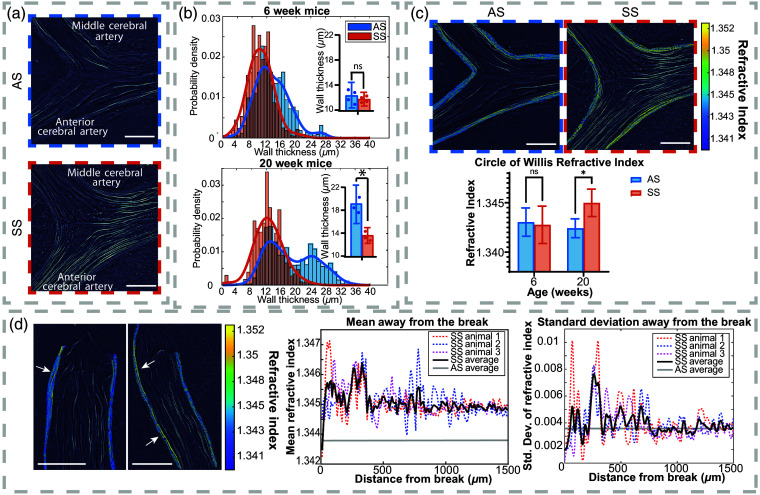
CoW blood vessel quantitative analysis. (a) Images of the blood vessel junction of the MCA and the ACA on the CoW. The top image (blue) is from an AS mouse, and the bottom image (red) is from an SS mouse. (b) Vessel wall thickness at 6-week-old (top) and 20-week-old (bottom). Here, it is clear that the AS has thicker vessel walls than the sickle cell model. The inset bar graphs show the per-animal averages and data points to visualize the differences between the AS and SS groups. (c) Examples of the MCA/ACA/ICA junction showing the lower average RI in the AS mouse brain (top left) and higher RI in the SS brain (top right). The graph also shows the higher RI of the sickle cell brain for the 20-week-old mice. (d) An analysis of the breaks in the CoW vessels, including two visualizations of breaks along the CoW (left), a graph showing the changing mean RI value as a function of distance from the break (center), and a graph showing the standard deviation as a function of distance from the break (right). Arrows in the left figure point to fragmented high-RI fibers seen along the breaking points. All scale bars are 100  μm.

We first focus on the CoW and MCA as these arteries are of major clinical importance in SCD due to their role in strokes.^21^^,^^22^ We analyzed arteries along the CoW, with images taken around the ACA/ICA/MCA junction; example images of this junction are seen in Fig. 3(a). We imaged ∼1500  μm in all directions along this junction, as seen in Fig. S2 in the Supplementary Material.

First, we measured the thickness of the blood vessel wall. Figure 3(b) shows histograms summarizing the measurements. Here, each histogram of the vessel wall thickness shows the wall thickness values for each animal combined into a single histogram. The animals within an experimental group did not display significant differences from one another, and the individual measurements per animal can be seen in the inset graph in Fig. 3(b). For the 6-week-old animals, the wall thickness difference between the SS and AS models was not statistically significant (p=0.38); however, for the 20-week-old animals, the SS mice exhibited significantly thinner vessel walls compared with AS (p=0.05). Interestingly, the 20-week-old AS animals exhibit a bi-modal distribution, whereas the SS animals show only one normal distribution, with a mean nearly identical to the lower group of the AS. This distribution held true for each individual animal (hence this is not an artifact resulting from a single or a few animals with anomalous behavior). This pattern potentially indicates a thickening of vessels occurring in the AS animals between the age of 6 weeks and 20 weeks that does not happen in the SS animals.

By further analyzing the relationship between blood vessel thickness and location along the MCA/ICA/ACA junction, we observed that the AS blood vessels were thicker along vessel branch points. The SS animals did not exhibit these thicker parts of the vessels at these junctions. Specifically, at the MCA/ICA/ACA junction, the AS vessels had significantly thicker walls (p=0.04) with an average thickness of 18 and 32  μm for the 6-week-old and 20-week-old animals, respectively. In contrast, the SS animals showed an average vessel thickness of 16  μm in both 6-week-old and 20-week-old mice. Thus, the bimodal distribution seen in Fig. 3(b) for the 20-week-old AS mice is a result of a thickening of vessels at branching points, which is absent in the SS mice. This location in the vasculature is also where many of the breakages occur. All 20-week-old SS animals exhibit a rupture occurring within 500  μm of the MCA/ICA/ACA junction. The areas with ruptures in the SS animals are located with thinner blood vessels than vessels with comparable locations in the AS animals (18  μm and 29  μm, p=0.04). As such, we hypothesize that the thinner blood vessel walls in an area of major arterial junctions increases the likelihood of breakages occurring. These results have not previously been seen in unaltered cerebral vasculature; however, they do follow the same trend as previously reported by Song et al. using fixed carotid arteries,^4^ where major, high-pressure SS arteries exhibit thinner walls in 20-week-old animals.

In continuing the analysis of the blood vessel walls, we analyzed the RI properties of the vessels. The RI corresponds to the relative speed of light in a material, but it is also linearly related to dry mass in a biological sample.^29^ Thus, we hypothesize that the RI can be affected by (and thus serve as an indirect indicator of) the vessel wall density, strength, and/or potential tension due to differences in wall biophysical composition. Here, we would expect that a blood vessel wall with a higher RI would be more rigid or potentially have higher tension. As Fig. 3(c) shows, the SS animals exhibited an overall higher RI in the blood vessel walls in 20-week-old mice (p=0.04) and had a higher degree of variation with a larger standard deviation (f-test p-value=0.04).

Another apparent difference between the SS and control AS groups was an increased number of blood vessel breakages in SS mice in both 6-week-old and 20-week-old mice. Half of the 6-week-old SS animals and all imaged 20-week-old SS animals exhibited at least one blood vessel break in the CoW, primarily along the ACA and MCA within 500  μm of the ACA/ICA/MCA junction. In comparison, none of the AS mice at either age group exhibited breakages along the CoW vessels.

Interestingly, we observed that the RI has high variance in areas near the breakages [Fig. 3(d)] with both local maxima and minima nearby in both the 6-week-old and 20-week-old mice. Figure 3(d) shows a 20  μm RI moving average and standard deviation graph comparing the RI as a function of the distance away from the break. For a single pixel in the image, the distance to the break was calculated as the distance along the blood vessel wall to the breakage in the vessel. Across the SS animals (AS animals did not exhibit breaks), three major regions were identified: (1) an area <50  μm from the break with a much lower mean RI (p=0.03) and a lower amount of variation (f-test p=0.02) than in the rest of the vessels of SS brains; (2) an area 50 to 350  μm from the break with a slightly increased mean RI but highly erratic variance; and (3) an area >350  μm from the break where the mean and standard deviation seem to stabilize and match the values of areas from the other side of the CoW that contains no breaks within the SS group. In the region with high amounts of variance located 50 to 350  μm from the break, the qOBM images showed local areas of extremely high RIs that were not seen elsewhere in the tissue nor in the AS animals [see arrows in Fig. 3(d)]. We also note that the mean value in the areas of high variance did not vary significantly from areas further away from the break (p=0.69).

These data suggest that the vessel walls of SS mice brains contain physiological differences at the tissue and cellular level affecting their RI properties. Although the results of this study cannot directly assess the reason for this change, we hypothesize that because the RI is proportional to mass density (and thus dry mass), such vessel RI changes can be an indicator of vessel rigidity, vessel strength, and/or tensions. This further suggests that these variables contribute to the breakages occurring in the CoW. Areas of higher RI may correspond to increased rigidity and/or tension. With compromised vessels, we expect that, under any sort of increased pressure (from SCD itself or from the perfusion process in these experiments), the thin, higher RI (more rigid and/or higher tension) vessels are more prone to breakages—especially at major arterial junctions. Future *in vivo* work may shed additional insight into the remarkable changes in the RI properties of vessels and how these changes progress with the disease using longitudinal scans of intact brains/vessels on the same animals over time.

### 3.2 SS Cortex Vessels Exhibited Greater Retention of Blood Cells and Higher RI Blood Vessel Walls

**Fig. 4:**
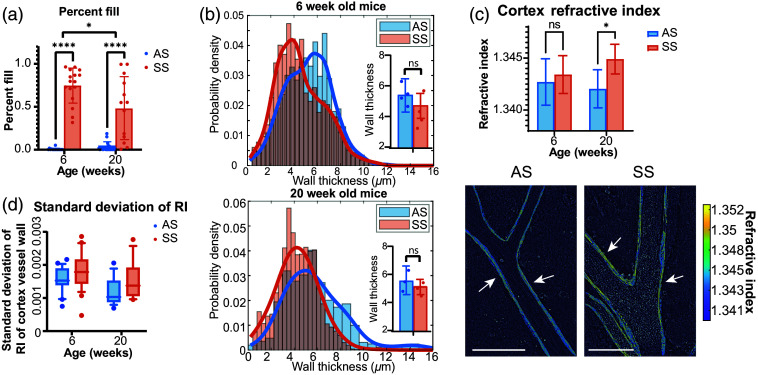
Cortex vessel quantitative analysis. (a) Percentage of blood vessels filled with blood cells shows a significant difference between sickle cell vessels versus AS vessels in 6-week-old and 20-week-old mice. (b) Histograms showing the distributions of the blood vessel wall thickness of the AS and SS mice in 6-week-old (top) and 20-week-old mice (bottom). These distributions do not show any statistically significant differences between the AS and SS animals. Though insignificant, one can see a skewness of the graphs showing AS animals with blood vessels with skewed thicknesses greater than those of the SS animals. The inset bar graphs show the per-animal averages and data points to visualize the differences between the AS and SS groups as well as inter-animal homogeneity. (c) (Top) a comparison of the RIs and (bottom) representative visualizations of the RI of an AS cortex vessel (left) versus an SS cortex vessel (right). Note the high RI fibers that can be seen and quantified in the AS and SS vessels as indicated by the white arrows. Scale bars are 100  μm. (d) Box and whisker plot to show the higher standard deviation observed in the SCD animals.

With statistically significant differences observed in the cerebral arteries of the CoW in the mouse brain, we then switched to cortex vasculature on the superior side of the brain that would be accessible in mice *in vivo*. Cortex vessels are smaller vessels that are downstream from the CoW vessels. Although we currently cannot differentiate between arteries and veins among the cortex vessels using qOBM, we wanted to assess if differences observed in CoW arteries would be comparable. Unlike the CoW, no vessel breakages were observed in the cortex; however, drastic differences were observed in the amount of blood cells remaining in the blood vessel between the SS and AS animals [example images seen in Fig. 2(b)]. The SS mice exhibited cortex blood vessels filled with blood cells, whereas the AS groups demonstrated a much lower fill fraction. All mice being perfused with heparinized saline using the same protocol suggests differences in RBC adherence in the smaller vessels or, if they were veins, that deoxygenation leads to these aggregations and accumulations in cortex vessels. SS mice contained a statistically significant (p<0.001) higher fraction of blood cells remaining in the vessels than in the AS mice for both 6-week-old and 20-week-old mice, with each data point representing an imaged cortex blood vessel [Fig. 4(a)]. The remaining blood cells in the cortex vessels could be a result of a vessel break preceding this location causing loss of pressure in the vessel and an incomplete flushing out of the blood cells. This also suggests that all SS animals at all ages either had vessel breakages somewhere in the CoW or could have developed aggregations and cell adhesions to the vessel wall, preventing the blood cells from being flushed out. Sickled RBCs in the deoxygenated conditions would exacerbate these blockages.

Next, we analyzed the cortex blood vessels wall thickness. For the 6-week-old mice, no statistically significant differences were observed between the AS and SS animals (p=0.43). Similarly, no statistical differences were observed for the 20-week-old mice (p=0.52), which is different from the thickness difference observed in the CoW. The difference between the two regions (cortex and CoW) can be attributed to the fact that there are higher pressures in the CoW arteries compared with cortex vessels; thus higher stress is placed on the CoW blood vessels.

The statistical significance above was assessed using the average values of each animal as an independent data point. However, if instead we take the average value of each observed blood vessel as an independent data point [histogram in Fig. 4(b)], we observe that the AS group has a slightly more skewed distribution with larger vessel walls. Assuming that each vessel can be treated as independent, a two-sided t-test reveals statistically significant differences for both the 6-week-old and 20-week-old animals (p=0.04 and p=0.05, respectively). Although this may be a weak assumption, it at least suggests that, with a larger population, we may be able to elucidate trends between the blood vessel wall thickness of AS and SS mice even outside the MCA/ICA/ACA junction. A further analysis of the statistical significance dependant on the independent data points can be seen in the Sec. 3 in the Supplementary Material.

Finally, we analyze the RI composition of the cortex vessels. In the 20-week-old mice, the SS mice vessel walls show a significantly higher RI (p=0.05); however, differences in the 6-week-old mice are not statistically significant (p=0.66), which is consistent with the CoW results. Furthermore, the SS vessel walls also contain a significantly greater amount of variation in the RI along the length of the vessels than in the AS vessels (f-test p=0.0184). These variations are plotted in Fig. 4(d), which represents the variation of the RI showing the greater variation of SS animals for both age groups. Figure 4(c) shows an example AS vessel with a lower RI and an SS vessel with a higher RI and larger variations. Areas of a higher RI in blood vessels may correspond, at least in part, to collagen and elastin fibers in the blood vessel walls, though further investigation is needed to verify composition and any quantitative relationship with the RI. Regardless, the AS group contains blood vessels with long, continuous, well-organized fiber-like structures that possess a high RI [see arrows in Fig. 4(c)]. The SS vasculature, on the other hand, shows fiber-like high RI structures that are more fragmented and disorganized and have a higher density. This result of fragmented elastin fibers has been previously shown in Song, et al., in the carotid arteries of SS mice.^4^ These structures are highlighted in Fig. 4(c) by white arrows. These data further suggest that the vessel walls of SS mice brains contain physiological differences at the cellular and sub-cellular level, affecting their RI properties. We hypothesize that the RI may be an indirect indicator of vessel rigidity, vessel strength, and/or tensions resulting from these cellular and tissue changes, and as our data show, these properties are changing over time for the SS animals.

## 4 Conclusion

In this work, we applied qOBM to study brain blood vessel structural and biophysical changes that occur due to SCD using whole, fresh, perfused mouse brains. qOBM provides unique high-resolution RI information of blood vessel completely label-free *in situ* with clear quantitative cellular and tissue level details. Results from this study show important differences between SS and AS mouse cerebrovasculature, including the thickness of blood vessel walls and the RI composition. We also noted a greater amount of blood cells remaining in the cortex vessels of SS mice despite perfusion, indicative of either RBC aggregations and adhesions or a loss of pressure in the vasculature. We hypothesize that differences in blood flow due to SCD affects vasculature strength, flexibility, and/or rigidity, which we see manifested as vessel breakages and changes in vessel thickness and RI composition. Importantly, we saw thinner blood vessel walls at major arterial bifurcation points along the CoW in the SS animals. These thinner blood vessels likely played a role in the ruptures observed nearby. At these ruptures, we also observed a unique behavior in the RI composition of vessels, with a lower RI being observed right at the breakage point, followed by highly erratic RI fluctuations and then a stabilization distance. This behavior is in addition to a globally higher RI value in the vessels of SS mice compared with the AS group in 20-week-old mice. Such behavior suggests that the RI may be related to (or indicative of) vessel rigidity, strength, and/or tension, in addition to dry mass and density. Further analysis is necessary to better understand these biophysical properties and their relationship, but to the best of our knowledge, this is the first time such behavior has been observed. Indeed, other label-free optical methods (e.g., QPI) have been applied to study SCD, but they have primarily focused on analyzing blood cells *in vitro*.^30^^–^^35^ One exception is a study by Kim et al.;^36^ however, to gain access to vessels and blood flow within *in vivo*, intestinal vessels had to be exteriorized and placed in a transmissive imaging chamber. The analysis presented here highlights the unique ability of qOBM to show detailed quantitative insight, with sub-cellular resolution, in thick scattering samples.

This work serves as a starting point for future studies to improve our understanding of the effects of SCD on the vasculature. Because qOBM is a label-free imaging technique that operates in epi-mode, it can be applied *in-vivo* to observe blood cell adhesion within vessels, aggregations, vessel ruptures, and repair. *In vivo* studies may also reveal mechanisms underlying the increase in the RI as the mice age. Through studying the mechanisms leading to stroke and vaso-occlusive crises events *in vivo*, we hope that qOBM may be able to provide unique insight into SCD mechanisms and potentially help monitor the efficacy of developing therapeutic treatments.

## Supplementary Material



## Code, Data, and Materials

Data and code underlying the results presented in this paper are not publicly available at this time but may be obtained from the authors upon reasonable request.
