# Supplementary material for: Analysis of structural effects of sickle cell disease on brain vasculature of mice using three-dimensional quantitative phase imaging
Source: J Biomed Opt. 2023 Sep 9;28(9):096501. doi: 10.1117/1.JBO.28.9.096501 (PMC10491933; doi:10.1117/1.JBO.28.9.096501)
Supplement: Supplementary file 1 [file JBO_028_096501_SD001.pdf]

**Supplemental Material**

## S.1. Analysis of Additional Image Features

Here we provide the results of additional image features analyzed in this work that were not statistically significant. These factors include the diameter of cortex and CoW vessels, the tortuosity of the cortex vessels, and the angle that the MCA intersects the CoW.

### Vessel Diameter

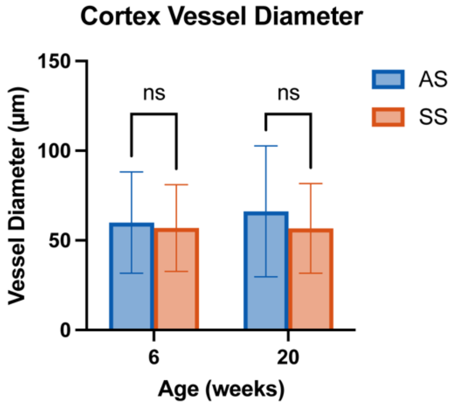


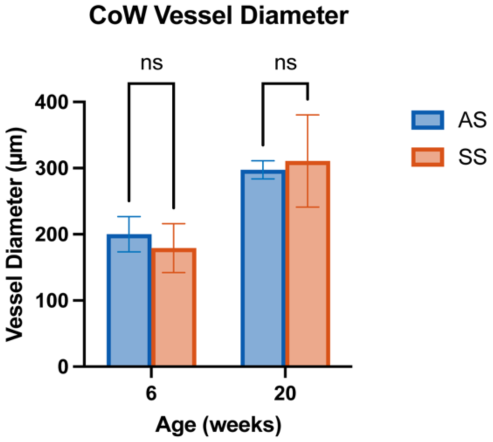


### Tortuosity

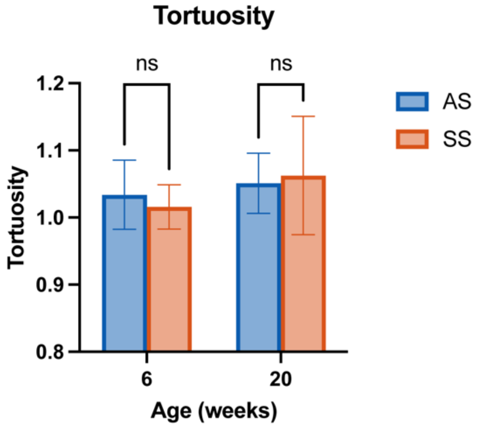


### MCA Angle

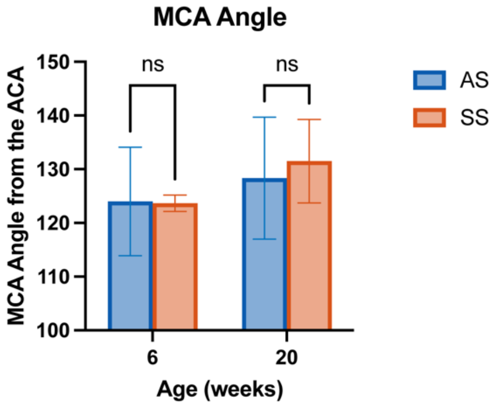

**S.2. Whole Circle of Willis Stitched Images**

The images below show stitched qOBM images of the Circle of Willis vessels.

AS 6 Week Vessels

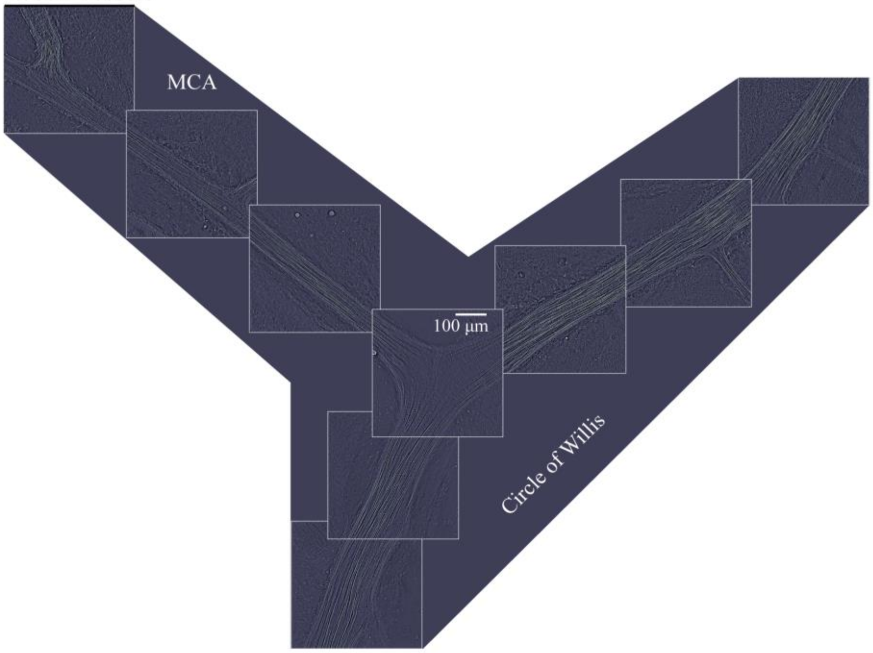


SS 6 Week Vessels

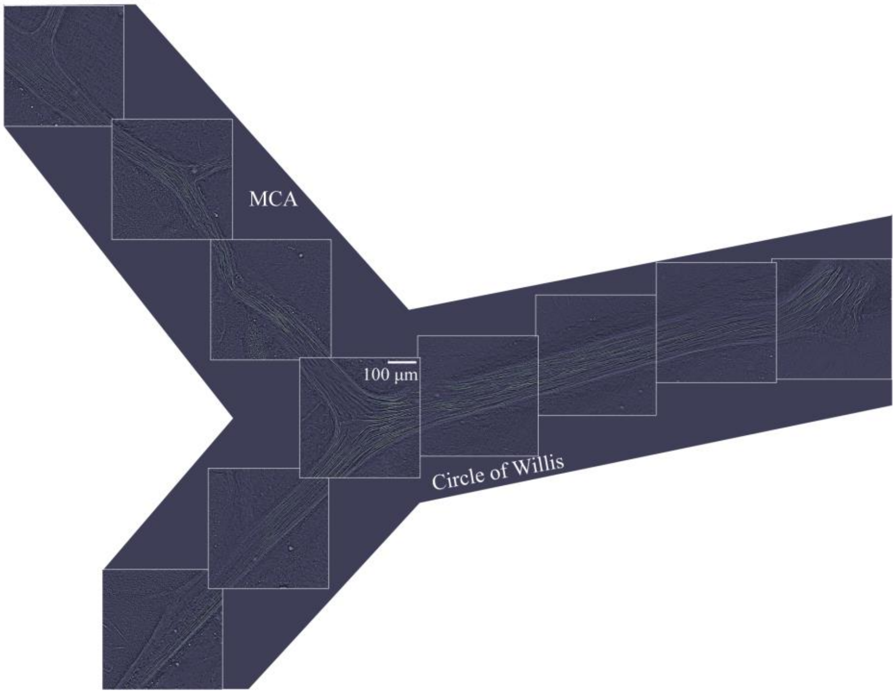

AS 20 Week Vessels

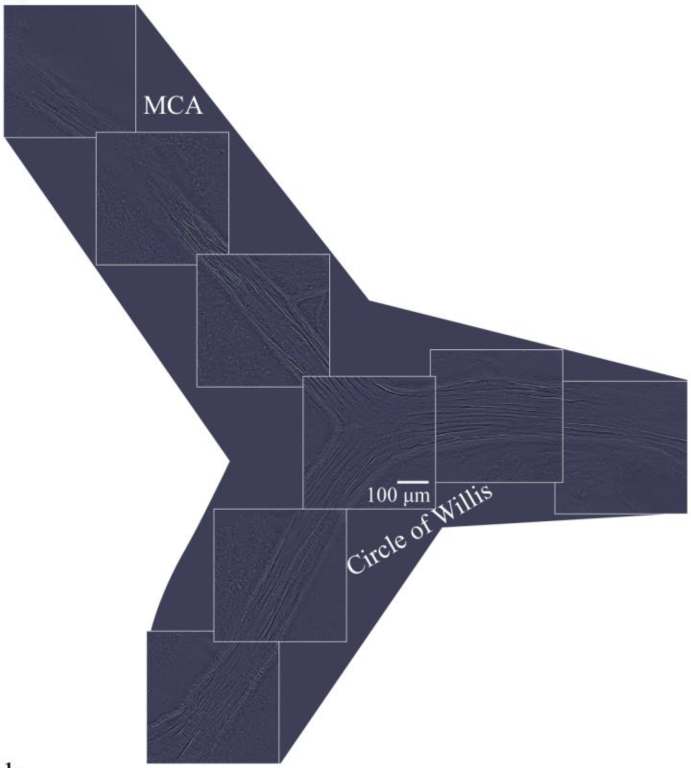


SS 20 Week Vessels

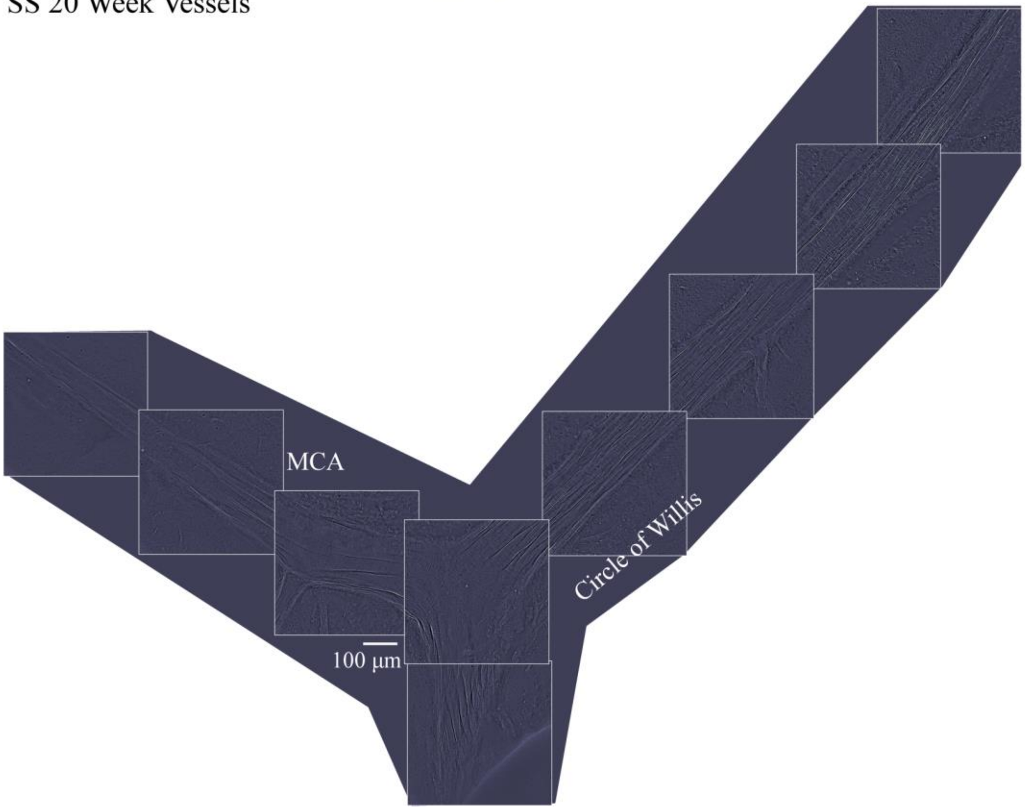

### S.3. Statistical Analysis

As discussed in the main text, statistical tests were calculated on a per animal basis. We also calculated the statistical significance for the cortex vessel assuming each imaged blood vessel was an independent measurement – thus increasing the effective sample size and increasing statistical significance. While this may be a weak assumption, it suggests that in future studies, with a larger number of animals, some of these parameters may show statistically significant differences between the control group and the SS disease groups. P-values of two-sided t-tests are provided in the table below.

| | *Per Animal Basis* | | *Per Vessel Basis* | |
|---|---|---|---|---|
| | *6 wk.* | *20 wk.* | *6 wk.* | *20 wk.* |
| *Cortex Wall Thickness* | 0.43 | 0.52 | 0.04 | 0.05 |
| *Cortex Refractive Index* | 0.66 | 0.05 | 0.64 | 0.02 |
| *Cortex Percent Fill* | <0.001 | <0.001 | <0.001 | <0.001 |
| *Cortex Vessel Diameter* | 0.89 | 0.68 | 0.76 | 0.34 |
| *Tortuosity* | 0.26 | 0.68 | 0.20 | 0.67 |
